# Prevalence and factors associated with adult diabetes mellitus in the adult population of Bunia, Democratic Republic of Congo: a cross-sectional study

**DOI:** 10.11604/pamj.2026.53.41.49576

**Published:** 2026-01-29

**Authors:** David Dyikpanu Tibasima, Yves Wasnyo, Ngundukali Ndenane, Joelle Laure Sobngwi-Tambekou, Philomène Mbassi Missi, Eugène Sobngwi

**Affiliations:** 1 Department of Public Health, School of Health Sciences, Catholic University of Central Africa, Yaoundé, Cameroon,; 2 Department of Epidemiology, Community Health, Nyankunde Higher Institute of Medical Techniques, Bunia, Democratic Republic of Congo,; 3 Department of Public Health, Faculty of Medicine and Biomedical Sciences, University of Yaoundé I, Yaoundé, Cameroon,; 4 *Recherche-Santé et Développement* (RSD Institute), Yaoundé, Cameroon,; 5 Department of Medical Biology and Laboratory, Nyankunde Higher Institute of Medical Techniques, Bunia, Democratic Republic of Congo,; 6 Departement of Nursing Sciences, School of Health Sciences, Catholic University of Central Africa, Yaoundé, Cameroon,; 7 Departement of Internal Medicine and Specialties, Faculty of Medicine and Biomedical Sciences, University of Yaoundé I, Yaoundé, Cameroon

**Keywords:** Diabetes mellitus, prevalence, associated factors, Bunia City

## Abstract

**Introduction:**

diabetes mellitus is a global public health emergency, with a worldwide prevalence of 11.1% and a 77.6% increase in mortality over the past decade. In Bunia, Ituri Province, persistent armed conflict and reliance on commercially imported foods may influence diabetes risk, warranting an assessment of its prevalence and associated factors.

**Methods:**

a community-based cross-sectional study was conducted in Bunia from March to July 2024 using multistage stratified random sampling. Adults aged ≥18 years who had lived in Bunia for at least six months were included. Data were collected through structured questionnaires and anthropometric, physical, and biochemical measurements following the WHO STEPwise approach. Descriptive, bivariate, and multivariate analyses were performed.

**Results:**

a total of 1,808 participants were enrolled, including 1,086 women (60.07%), with a mean age of 39.95 ± 17.01 years. Secondary education was reported by 54% of participants, 54.81% were married, and 25.66% were engaged in commercial activities. Monthly income below USD 100 was reported by 43.53%. The prevalence of diabetes was 6.31% (95% CI: 5.2-7.5), and prediabetes was 12.17% (95% CI: 10.7-13.8). Diabetes prevalence was higher among women (4.03%) than men (2.27%). Factors independently associated with diabetes included advanced age (aOR = 1.029; 95% CI: 1.011-1.047), abdominal obesity (aOR = 1.035; 95% CI: 1.015-1.056), prolonged sedentary behavior (aOR = 1.002; 95% CI: 1.001-1.003), low-intensity physical activity (aOR = 2.002; 95% CI: 1.053-3.813), semi-urban residence (aOR = 2.655; 95% CI: 1.381-5.106), and occupations such as farming or mason´s assistant (aOR = 2.718; 95% CI: 1.084-6.814). Protective factors included family history of diabetes (aOR = 0.337; 95% CI: 0.204-0.558), appropriate salt intake (aOR = 0.527; 95% CI: 0.295-0.943), and low stress levels.

**Conclusion:**

diabetes prevalence in Bunia is shaped by demographic, behavioral, occupational, and environmental factors, underscoring the need for targeted prevention, health promotion, and management strategies for high-risk populations.

## Introduction

Diabetes mellitus is a chronic metabolic disorder characterised by persistent hyperglycaemia resulting from insufficient insulin secretion or activity [1]. It is a major global public health issue [2] because of its increasing prevalence and the severe complications associated with it [3]. These complications affect various organs and significantly increase the risk of premature death [2,4]. In 2019, approximately 4.2 million adults aged 20-79 years are estimated to have died from diabetes and its complications, which equates to almost one death every eight seconds [3]. Beyond this medical burden, the disease also leads to significant psychosocial difficulties, such as denial, social isolation, stigma, the constraint of lifelong treatment, and the necessity to follow stringent hygiene and dietary recommendations [5]. These factors have direct and indirect impacts on the quality of life of those affected and their healthcare expenditures [6,7].

Globally, the prevalence of diabetes continues to increase [8]. The International Diabetes Federation estimates that 11.1% of the adult population currently lives with the disease, a figure that could reach 13% by 2050 [9]. This increase is driven by population aging, nutritional transition, rapid urbanisation, and the adoption of sedentary lifestyles that promote obesity [8,10]. In Africa, the situation is particularly concerning, with an estimated increase in prevalence of over 85% between 1990 and 2019 [9]. However, the data remain heterogeneous across countries, with overall prevalence rates ranging from 2.6% to 20% [11]. In the Democratic Republic of the Congo, several local studies have reported rates between 3.5% and 6.7%, depending on the contexts studied [12-14]. However, no national survey has been conducted, and the available data are often limited to specific rural or urban areas, making it difficult to provide a representative estimate of the national situation in this regard.

Bunia, the capital of the Ituri Province, presents a particular socio-health context marked by recurrent armed conflicts, chronic psychosocial stress, massive population displacement, and accelerated urbanisation. These conditions promote rapid dietary habit changes, the introduction of imported foods, and lifestyle shifts, which are likely to increase the risk of metabolic diseases. A hospital study at the General Hospital of Bunia reported a diabetes prevalence of 4.38% [15]; however, data from a single institution do not reflect the actual situation within the general population.

In this context, reliable population data are necessary to guide local prevention and management strategies. Thus, this study aimed to determine the prevalence of diabetes and identify its associated factors in the adult population of Bunia.

## Methods

**Study design and context:** a cross-sectional study was conducted to determine the prevalence of diabetes mellitus and its associated factors among individuals aged ≥18 years living in the city of Bunia. The capital of the Ituri Province in North Eastern Democratic Republic of Congo, Bunia, comprises three communes (Mbunya, Nyakasanza, and Shari), subdivided into 24 neighbourhoods and 538 avenues [16]. It is characterised by three morphological zones: urban, semi-urban and rural. The city, which covers approximately 830 km^2^ [17], is located on the Ugandan border and falls within the Bunia health zone, equipped with a general referral hospital, 14 additional hospitals, and 17 health centres [16]. However, diabetes care remains inadequate, with only 25% of healthcare facilities providing dedicated services and a shortage of trained staff and essential medicines [18]. The preparation for the survey, including the selection and training of surveyors, community mobilisation, and public awareness among local authorities, took place from 1^st^ February to 27^th^ March, 2024 followed by a pilot survey on 22^nd^ and 23^th^ March, 2024. The main field survey will be conducted from 28^th^ March to 28^th^ June, 2024.

**Study population:** the study population consisted of adults who had been residing in Bunia for at least six months, were 18 years of age or older, and provided informed consent. The exclusion criteria were as follows: participants who withdrew their consent, pregnant women followed for their pregnancy, individuals who submitted incomplete questionnaires, and those with blood glucose levels ≥7 mmol/L who did not undergo confirmatory glycaemic testing. The minimum sample size was estimated using the Schwartz formula for cross-sectional studies [19].


$$n=\frac{{\left(Z\alpha \right)}^{2}p\left(1-p\right)}{e^{2}}$$

Where n represents the minimum sample size, Zα the coefficient corresponding to the desired confidence level (value of the x-coordinate of the standard normal distribution), p the estimated prevalence of the event under study in the target population, and e the margin of error or desired level of precision for estimating this prevalence. In practice, the value of e is generally between 1% and 10% depending on the objectives and feasibility of the study.

For this study, a 95% confidence level was used, corresponding to a Z-value of 1.96. The expected prevalence of diabetes was set at 5.8%, in line with estimates reported for the Democratic Republic of Congo by the International Diabetes Federation. An absolute precision of 1.3% (e=0.013) was chosen to obtain a sufficiently reliable estimate for the study population. Based on these parameters, the minimum sample size required was estimated at 1,242 participants. A multi-stage stratified sampling method was applied: the three zones of Bunia (urban, semi-urban, and rural) were first selected, followed by the random selection of 12 neighbourhoods using Excel´s “random between bounds” function. Thus, three, five, and four neighbourhoods were selected from the urban, semi-urban, and rural zones, respectively, for the study. The selected neighbourhoods were Lumumba, Bankoko, Yambi Yaya, Bakongolo, Kindia, Bigo, Ndibé, Simbilyabo, Dhélé, Kasegwa, Bembey, and Nyakasanza. In each of these, a systematic sampling method was used to select avenues and then households: the avenues were numbered, and every other avenue was included until approximately 100 participants per neighbourhood were recruited. The same procedure was applied to select households within each avenue.

### Data collection

**Data collection tools and instruments:** in this study, field data collection relied on a structured questionnaire adapted from the STEPS model of the World Health Organization (WHO) [20]. This tool, recognised for its standardisation and methodological validity in epidemiological surveys on non-communicable diseases, was chosen because of its relevance in assessing the prevalence of diabetes and its associated factors. The electronic questionnaire was designed in KoboToolbox and administered using the KoboCollect application on tablets. Nine tablets were used, three for each survey team. To address power supply issues in certain areas, three external batteries (one per team) were provided to ensure the continuity and reliability of the data collection process. Biometric and clinical measurements were performed using appropriate validated equipment. Weight was measured using five electronic adult scales, while height and waist circumference were measured using three 150 cm graduated measuring tapes. Blood pressure and heart rate were measured using five electronic sphygmomanometers. Fasting capillary blood glucose was measured using three G200 glucometers, accompanied by twenty boxes of G200 test strips (each containing 100 units) and twenty boxes of lancets of the same model (each containing 100 units). Capillary samples required sterile swabs, single-use sterile gloves, hydroalcoholic solutions, and vials of 70% denatured alcohol for disinfection. Three sterile drums were used to store the prepared swabs. To ensure the quality and safety of the survey process, the prepared swabs were systematically prepared and sterilised at the Higher Institute of Medical Techniques (ISTM) of Nyankunde in Bunia.

**Overall data collection process:** data collection was preceded by obtaining authorisation from local authorities and approval from relevant ethics committees. A team of 13 people was recruited, consisting of eight nurses, three medical biologists, one community development expert, and one economist. Among the eight nurses, five had completed postgraduate training in community health, four specialising in epidemiology and one in health and community development. Twelve community liaisons, one for each selected neighbourhood, were also chosen to prepare the households and assist the investigators in the relevant neighbourhoods and avenues. The team received two days of training, followed by two days of pilot surveys in three neighbourhoods not included in the study. The fieldwork was carried out in four successive stages, each covering the following neighbourhoods: Bakongolo, Kindia, and Nyakasanza 1; Bigo, Dhélé, and Bembey; Simbilyabo, Kasegwa, and Yambiyaya; and Lumumba, Bankoko, and Ndibé. On the eve of each visit, community representatives raised awareness among households. Each morning, the investigators, divided into three teams of four, checked the participants´ eligibility, obtained their informed consent (written and oral), and administered the questionnaire on a tablet via KoboCollect. Simultaneously, physical, anthropometric, and biochemical measurements were taken using standardised protocols: height while standing, barefoot; weight using an electronic scale; waist circumference measured between the last floating rib and the iliac crest; blood pressure taken after five minutes of rest, with three readings and the average used; fasting capillary blood glucose measured with a glucometer, with a second confirmatory reading if the value was ≥7 mmol/L. All data (questionnaire, physical, and biochemical measurements) were entered into KoboCollect and were transmitted daily to the KoboToolbox server. The principal investigator provided daily supervision, checked the data quality, and corrected anomalies in collaboration with the team leaders.

**Definitions:** sociodemographic variables (age, sex, marital status, ethnic origin, occupational category, education level, religious affiliation, area of residence, municipality, and income), behavioural variables (diet, alcohol consumption, smoking, and sedentary lifestyle), and anthropometric and clinical variables (BMI, waist circumference, weight, height, blood pressure, family history of diabetes, and diabetes status) were considered in this study. Diabetes mellitus was defined according to the following criteria: previous diagnosis of diabetes or fasting blood glucose ≥126 mg/dl (≥7.0 mmol/l) on two occasions among individuals unaware of their blood glucose status [1,3,21-23]. Prediabetes was defined as a blood glucose level between 6.1 and 6.9 mmol/l (110-125 mg/dl) [3]. Hypertension was defined as a systolic blood pressure ≥140 mmHg and/or diastolic blood pressure ≥90 mmHg, or a known history of hypertension [24-27]. Overweight was defined as a BMI between 25 and 29.9 kg/m^2^ and obesity as a BMI ≥30 kg/m^2^ [27]. Waist circumference was measured using a measuring tape, and abdominal obesity was defined as a waist circumference of ≥94 cm in men and ≥80 cm in women [21].

**Statistical analysis:** two main software programs were used in this study. Descriptive analyses were performed with Jamovi (version 2.3.28), while univariate and multivariate logistic regressions were carried out using R (version 4.5.0). Descriptive, univariate, and multivariate analyses were performed. Descriptive analysis was used to describe the characteristics of the study participants. Quantitative variables were summarised using the mean and standard deviation when they followed a normal distribution, and by the median as well as the first and third quartiles in the case of a skewed distribution. The normality of the distributions was assessed using box plots and the Kolmogorov-Smirnov and Shapiro-Wilk tests. Categorical variables were expressed as frequencies and percentages. In the univariate analysis, univariate logistic regression was used to examine the association between each explanatory variable and the study´s binary dependent variable (diabetes status). Crude odds ratios, 95% confidence intervals, and p-values were calculated. Multivariate logistic regression was performed to identify factors independently associated with diabetes. Several explanatory variables were simultaneously included in the model to assess their adjusted association with diabetes status. The results are presented as adjusted odds ratios (ORs) with 95% confidence intervals (CIs) and p-values. All variables found to be significant in the univariate regression, as well as those recognised in the literature as potential factors associated with diabetes and with a p-value ≤0.25 [28], were included in the multivariate model. Nagelkerke R^2^ was calculated to estimate the effect size and overall strength of the association of the model. To assess the associations between the covariates and the dependent variable, diabetes mellitus, we considered a significance threshold of 5% for the p-value, odds ratios (ORs) as approximations of the relative risk, and their 95% confidence intervals (95% CI). An odds ratio of 1 indicated no association between the independent variables and diabetes. An odds ratio between 0 and 1 suggests a protective factor, whereas an odds ratio greater than 1 indicates a risk factor [28]. The association between the covariates and the variable of interest was considered significant if the following conditions were met: OR >1 for a risk factor, p-value <0.05, and the 95% confidence interval for the OR did not include the value of 1 [28].

**Ethical considerations:** before proceeding with the survey, both oral and written consent were obtained from each participant. A consent form was prepared for this purpose. In the context of this study, the protection of personal data was ensured. To guarantee privacy and confidentiality, access to the survey data was limited to researchers and participants only. Identification codes were assigned to the participants to ensure that their names did not appear on the questionnaires. The investigators were also made aware of the ethical aspects of the study. This study was approved by the Institutional Ethics Committee for Human Health Research (CEIRSH) of the Faculty of Health Sciences of the Catholic University of Central Africa (ethical authorisation no. 2023/20499/CEIRSH/ESS/MSP) and the Research Ethics Committee of the Higher Institute of Medical Techniques of Nyankunde in Bunia (ethical authorisation no. 001/2024). It also received research authorisation (no. 054/086/DPS/IT/03/2024) was issued by the head of the Ituri Provincial Health Division and approved by the provincial and municipal authorities.

## Results

**General characteristics of the studied population:** this section presents the sociodemographic, behavioural, and clinical characteristics of the participants included in the study conducted in Bunia on the prevalence of diabetes mellitus and its associated factors. In total, 1,808 participants were surveyed, of whom 1,086 (60.07%) were women. The average age was 39.95 ± 17.01 years. The majority had a secondary education level (973, 53.82%), were married (991, 54.81%), and belonged to the Protestant denomination (892, 49.34%), followed by Catholics (651, 36.01%). Nearly half of the respondents resided in the Mbunya municipality (870, 48.12%). The most represented ethnic group was the Hema (655, 36.23%), and merchants made up 25.66% of those surveyed. In addition, 787 participants (43.53%) reported a monthly income of less than 100 dollars.

On the behavioural level, approximately 5% of respondents were smokers at the time of the survey, and 12% reported exposure to passive smoking. Regarding alcohol consumption, 471 (26.05%) had consumed alcohol before, of whom 295 (16.32%) were still consuming it, with approximately 23% drinking daily. High-intensity physical activity was practiced by 543 subjects (30.03%) and moderate-intensity activity by 364 (20.13%). Only 11.73% of the participants engaged in sports. Walking was the main mode of transportation (67.53%), followed by the use of motorcycles (28.76%).

Clinically, the median waist circumference was 86 cm (78-95) for women and 81 cm (75-89) for men. The median fasting blood glucose level at the first sampling was 5.2 mmol/L (4.7-5.8). Among participants with blood glucose >7 mmol/L, the median at the second sampling was 6.3 mmol/L (4.9-8.2 mmol/L), with the overall median remaining at 5.2 mmol/L (4.7-5.8 mmol/L). The median systolic and diastolic blood pressures were 124 mmHg (112-139) and 79 mmHg (71-89), respectively. The median body mass index was 23.5 kg/m^2^ (21.1-26.6).

The overall prevalence of diabetes mellitus was 6.31% (95% CI: 5.2-7.5), including 4.70% of known cases and 1.60% of newly diagnosed cases. Among the individuals with diabetes, 73 (4.03%) were women, and 43 (2.27%) were men. Additionally, 220 participants (12.17%) were diagnosed with prediabetes. The average age at diagnosis was 51.6 ± 13.6 years (52.6 ± 12.4 years for previously known cases and 49 ± 16.5 years for new cases).

**Univariable analysis:** in the univariate analysis, several factors were significantly associated with diabetes: age (OR = 1.054; 95% CI: 1.043-1.066; p < 0.001), level of education, marital status, municipality of residence, area of origin, occupation, ethnicity (Table 1), amount of salt consumed, means of mobility used, daily time spent sitting or lying down (OR = 1.129; 95% CI: 1.070-1.191; p < 0.001), exposure to stress, frequency of vegetable consumption (OR = 1.204; 95% CI: 1.058-1.369; p = 0.005) (Table 2), family history of diabetes, arterial hypertension, and abdominal circumference (Table 3).

**Table 1:** univariable and multivariable logistic regression of sociodemographic factors associated with diabetes mellitus in the city of Bunia, Democratic Republic of Congo, from March to July 2024 (n=1808)

Variable	Unadjusted ORs (95% CI)	p-value	Adjusted ORs (95% CI)	p-value
Age in years	1.054 (1.043-1.066)	< 0.001	1.029 (1.011-1.047)	0.002
**Sex**				
Male	Ref			
Female	1.19 (0.81 - 1.78)	0.37		
**Education levels**				
Secondary	Ref		Ref	
No instructions	1.85 (0.91-3.75)	0.09	1.31 (0.536-3.119)	0.554
Primary	2.03 (1.32 - 3.15)	0.001	1.112 (0.626-1.975)	0.718
Superior	0.87 (0.48 - 1.58)	0.65	0.724 (0.320-1.638)	0.438
**Marital status**				
Widowed	Ref		Ref	
Bachelor	0.06 (0.025 - 0.15)	< 0.001	0.516 (0.167-1.597)	0.251
Married	0.42 (0.27 - 0.66)	< 0.001	1.181 (0.526-2.229)	0.608
Divorced	0.33 (0.11 - 0.97)	0.04	0.855 (0.249-2.941)	0.804
**Religious affiliation**				
Protestant	Ref			
Catholic	0.97 (0.65 - 1.45)	0.88		
Revival church	0.37 (0.13 - 1.03)	0.057		
Muslim woman	0.74 (0.26 - 2.10)	0.58		
Other (please specify)	/	0.99		
**Municipalities**				
Shari	Ref		Ref	
Mbunya	1.03 (0.66 - 1.61)	0.91	0.859 (0.407-1.812)	0.69
Nyakasanza	2.12 (1.28 - 3.51)	0.004	0.808 (0.361-1.805)	0.603
**Areas of belonging**				
Urban trend	Ref		Ref	
Semi-urban	2.80 (1.76 - 4.45)	< 0.001	2.655 (1.381-5.106)	0.003
Rural trend	1.04 (0.57 - 1.90)	0.9	1.204 (0.519-2.794)	0.665
**Ethnic groups**				
Sudanese	Ref			
Bantu	0.69 (0.40 - 1.18)	0.176		
Nilo-hamite	0.71 (0.42 - 1.19)	0.195		
**Ethnicities**				
Lendu	Ref		Ref	
Alur	0.73 (0.35 - 1.51)	0.399	0.809 (0.335-1.959)	0.639
Bira	0.57 (0.26 - 1.25)	0.162	0.491 (0.192-1.253)	0.137
Hema	0.49 (0.267 - 0.917)	0.025	0.48 (0.212-1.087)	0.078
Other ethnic groups of Ituri	0.266 (0.095 - 0.744)	0.012	0.365 (0.111-1.206)	0.098
Ethnic groups of Kivu or Maniema	0.81 (0.38 - 1.72)	0.583	1.327 (0.495-3.557)	0.574
Other ethnic groups in the DRC	0.476 (0.228 - 0.99)	0.047	0.744 (0.296-1.872)	0.53
**Monthly income**				
Greater than or equal to $1100	Ref			
Less than $100	0.547 (0.188 - 1.585)	0.266		
From $100 to $299	0.530 (0.176 - 1.597)	0.259		
From $300 to $499	0.316 (0.067 - 1.492)	0.146		
From $900 to $1099	/	0.999		
**Professional activities**				
Nothing	Ref		Ref	
Government employee	1.264 (0.510 - 3.136)	0.613	1.553 (0.493-4.892)	0.452
Merchant	1.172 (0.633 - 2.169)	0.614	1.284 (0.586-2.813)	0.532
Farmer or stonemason's helper	1.611 (0.818 - 3.171)	0.168	2.718 (1.084-6.814)	0.033
Teacher or Minister of Religion	2.471 (1.158 - 5.273)	0.019	2.809 (0.993-7.951)	0.052
Health service personnel	2.044 (0.771 - 5.422)	0.151	2.358 (0.686-8.110)	0.173
Other (please specify)	0.867 (0.456 - 1.650)	0.664	1.380 (0.599-3.180)	0.449

**Table 2:** univariable and multivariable logistic regression of behavioral factors associated with adult diabetes mellitus in the city of Bunia, Democratic Republic of Congo, from March to July 2024 (n=1808)

Variable	Unadjusted ORs (95% CI)	p-value	Adjusted ORs (95% CI)	p-value
**Active smoking**				
Yes	Ref			
No	2.922 (0.710-12.031)	0.138	2.166 (0.452-10.375)	0.334
**Passive smoking**				
Yes	Ref			
No	1.44 (0.74 - 2.80)	0.283		
**Current alcohol consumption**				
Yes	Ref			
No	1.043 (0.620 - 1. 753)	0.875		
**Past alcohol consumption**				
Yes	Ref			
No	0.749 (0.497 - 1.128)	0.166	0.735 (0.444-1.216)	0.23
**Fruit consumption**				
Yes	Ref			
No	0.935 (0.639 - 1.369)	0.73		
Weekly fruit consumption frequency	0.990 (0.838-1.169)	0.903		
Daily fruit portion	0.877 (0.667-1.154)	0.348		
**Adding salt to food**				
Always	Ref		Ref	
Never	1.516 (0.349 - 6.575)	0.578	1.015 (0.164-6.269)	0.987
Sometimes	0.450 (0.096 - 2.108)	0.311	0.546 (0.085-3.491)	0.523
Rarely	0.637 (0.144 - 2.809)	0.551	0.533 (0.087-3.261)	0.496
Often	0.319 (0.061 - 1.667)	0.175	0.286 (0.040-2.028)	0.21
**Quantity of salt consumed**				
Too little	Ref		Ref	
Far too much	0.643 (0.192- 2.154)	0.474	0.247 (0.061-1.000)	0.05
Much too little	1.606 (0.906-2.848)	0.105	0.958 (0.466-1.972)	0.908
Just the right amount	0.368 (0.226-0.601)	< 0.001	0.527 (0.295-0.943)	0.031
Does not consume salt	3.147 (1.647-5.917)	< 0.001	1.048 (0.460-2.387)	0.911
Don't know	0.560 (0.130-2.408)	0.436	0.453 (0.091-2.246)	0.332
Too much	0.352 (0.107-1.165)	0.087	0.623 (0.150-2.595)	0.516
**High-intensity physical activity**				
Yes	Ref		Ref	
No	1.487 (0.948-2.331)	0.084	2.003 (1.053-3.813)	0.034
**Moderate-intensity physical activity**				
Yes	Ref		Ref	
No	1.584 (0.921-2.721)	0.096	0.589 (0.159-2.184)	0.428
**Common mode of transportation**				
Car or other vehicle	Ref		Ref	
Walking	0.377 (0.154-0.921)	0.032	1.474 (0.429-5.061)	0.537
Motorcycle	0.469 (0.186-1.183)	0.109	1.666 (0.485-5.720)	0.418
Other	3.250 (0.883-11.967)	0.076	3.133 (0.543-18.058)	0.201
**Sport practice**				
Yes	Ref			
No	2.104 (0.966-4.584)	0.061	0.658 (0.118-3.667)	0.633
Duration of sport sessions	0.231 (0.052-1.030)	0.055	0.996 (0.986-1.007)	0.479
Weekly frequency of sport	0.990 (0.558-1.754)	0.972		
Time spent sitting or lying down (in hours)	1.129 (1.070-1.191)	< 0.001	1.002 (1.001-1.003)	0.017
Always	Ref		Ref	
Never	0.181 (0.041-0.795)	0.024	0.306 (0.059-1.590)	0.159
Sometimes	0.389 (0.212-0.716)	0.002	0.359 (0.167-0.772)	0.009
Rarely	0.297 (0.143-0.616)	0.001	0.365 (0.143-0.926)	0.034
Often	0.805 (0.462-1.403)	0.445	0.741 (0.371-1.483)	0.397
Weekly frequency of vegetable consumption	1.204 (1.058-1.369)	0.005	1.114 (0.953-1.304)	0.176

**Table 3:** univariable and multivariable logistic regression of clinical factors associated with adult diabetes mellitus in the city of Bunia, Democratic Republic of Congo, from March to July 2024 (n=1808)

Variable	Unadjusted ORs (95% CI)	p-value	Adjusted ORs (95% CI)	p-value
**Family history of diabetes mellitus**				
Yes	Ref		Ref	
No	0.404 (0.269-0.607)	<0.001	0.337 (0.204-0.558)	<0.001
**High blood pressure**				
Yes	Ref			
No	0.243 (0.161-0.368)	<0.001	0.726 (0.430-1.223)	0.229
Waist circumferences (in cm)	1.048 (1.036-1.060)	<0.001	1.035 (1.015-1.056)	0.001
BMI	1.090 (1.057-1.124)	<0.001	0.989 (0.935-1.046)	0.704
BMI: body mass index				

**Multivariable analysis:** in the multivariate logistic regression analysis, several factors were independently associated with diabetes among adults in Bunia. Increasing age significantly increased the risk of developing HCC (AOR = 1.029; 95% CI: 1.011-1.047; p = 0.002). Living in a semi-urban area was also a significant predictor (AOR = 2.655; 95% CI: 1.381-5.106; p = 0.003), as was having occupations such as farmer or masonry assistant (AOR = 2.718; 95% CI: 1.084-6.814; p = 0.033) (Table 1). Salt consumption considered “just necessary” appeared to be a protective factor (AOR = 0.527; 95% CI: 0.295-0.943; p = 0.031) (Table 2). An insufficient level of intense physical activity increased the risk of diabetes (AOR = 2.002; 95% CI: 1.053-3.813; p = 0.034), as did the daily duration spent sitting or lying down (AOR = 1.002; 95% CI: 1.001-1.003; p = 0.017) (Table 2). Conversely, occasional (AOR = 0.359; 95% CI: 0.167-0.772; p = 0.009) or rare exposure to stress (AOR = 0.365; 95% CI: 0.143-0.926; p = 0.034) was associated with a lower probability of developing diabetes (Table 2). No family history of diabetes was also a significant protective factor (AOR = 0.337; 95% CI: 0.204-0.558; P < 0.001), whereas increased waist circumference was associated with a higher risk of diabetes (AOR = 1.035; 95% CI: 1.015-1.056; P = 0.001) (Table 3).

Strong collinearity was found between the municipality of residence and the area of origin in the multivariate analysis. Consequently, the “municipality of residence” variable was removed from the model to avoid distortions related to this collinearity and to improve the stability of estimates. The final model had a Nagelkerke R^2^ coefficient of 0.355, indicating that 35.5% of the variance in the diabetic status was explained by the variables included in the analysis.

## Discussion

This study evaluated the prevalence and factors associated with diabetes mellitus among adults in the city of Bunia, located in the Ituri Province in the North East of the Democratic Republic of the Congo. The prevalence of diabetes mellitus in Bunia was 6.31%, whereas 12.17% of the participants had prediabetes. This prevalence is slightly higher than that observed in the slums of Nairobi, Kenya [29]. However, it remains slightly lower than those reported by Mayega *et al*. in Uganda (7.4%) [30] and Muyer *et al*. in Kinshasa (6.7%) [12]. Regarding prediabetes, the prevalence observed in the present study was slightly higher than that reported by Peer *et al*.in South Africa (11.2%) [31]. The prevalence of undiagnosed or newly diagnosed diabetes in Bunia was 1.6%, slightly higher than that observed in South Africa (1.2%) [31], but lower than that reported in Ethiopia by Worede *et al*. (2.3%) [32]. These variations in prevalence may be explained by methodological, sociodemographic, and behavioural differences, as well as the level of urbanisation and access to healthcare services. The relatively high proportion of prediabetes observed in this study highlights an important reservoir of subjects at risk of progressing to overt diabetes, underscoring the need for early preventive interventions.

Regarding the factors associated with diabetes, both univariate and multivariate analyses revealed a significant association between age and diabetes in adults. In Bunia, older individuals were more frequently affected than younger ones. This result is consistent with those of other studies [31-34] and confirms the data in the literature, indicating that the prevalence of diabetes increases with age [27,35,36]. This association is explained by the gradual decrease in insulin sensitivity, accumulation of metabolic risk factors, and prolonged exposure to harmful behaviours over time.

Overweight and obesity are recognised as major risk factors for diabetes mellitus [27,35,36], as is high blood pressure [37]. In the present study, univariate analysis showed an association between these variables and diabetes. However, these associations were not maintained in the multivariate analysis, suggesting the effect of confounding factors or interdependence between certain variables, particularly abdominal obesity. Waist circumference emerged as one of the main factors independently associated with diabetes mellitus in Bunia. This result is consistent with studies conducted by Oraii *et al*. in Tehran in 2022 and Mohamed *et al*. in Kenya in 2018, which established a close link between abdominal obesity and diabetes mellitus [33,38]. Visceral fat accumulation is a determining factor of insulin resistance. In the sociocultural context of Bunia, the belief that abdominal obesity is an indicator of wealth and family well-being may contribute to the persistence of this risk factor for chronic diseases.

Physical inactivity is also recognised as a risk factor for diabetes mellitus [1,35,37]. Several studies have supported this relationship. Indeed, Atrese *et al*. in Ethiopia and Sarah K *et al*. in Kenya showed that a sedentary lifestyle is a significant risk factor for diabetes [39,40]. The results of the present study also show that people with low physical activity or who spend prolonged periods sitting or lying down were more exposed to diabetes in Bunia. This observation reflects the epidemiological transition observed in African urban areas, marked by a reduction in physical activity and an increase in sedentary behaviour.

This study also showed that a family history of diabetes was strongly associated with the occurrence of diabetes in the Bunia population. This result is consistent with those reported by Atrese *et al*. and Tesfaye *et al*. in Ethiopia [39,41]. This association reflects both the influence of genetic factors and the sharing of behaviours and environments within families that are conducive to the development of diabetes mellitus. These findings support targeted screening of individuals with a family history of the disease.

According to this study, diabetes was inversely associated with adequate salt consumption and low-to-moderate stress levels. Although excessive salt intake is not recognised as a direct risk factor for diabetes, it is strongly associated with high blood pressure, which is itself a factor frequently linked to diabetes within the context of metabolic syndrome.

Urbanisation is associated with an increased prevalence of diabetes mellitus [11,42,43]. However, the results of this study highlighted an association between diabetes and periurban areas. Several hypotheses can explain this finding. Downtown Bunia is dominated by commercial and administrative activities and has relatively fewer residential buildings. Moreover, a portion of its inhabitants belongs to a formerly wealthy population that is better informed about chronic diseases, which encourages the adoption of preventive behaviours. In contrast, peri-urban areas are home to an economically active population that is often exposed to precarious living conditions, an unbalanced diet, low levels of physical activity, and sometimes the indirect consequences of armed conflicts, all of which promote the adoption of unhealthy lifestyles.

The univariate analysis in this study showed an association between diabetes mellitus and the professions of teaching and religious leadership (pastors and priests). Regarding religious leaders, this finding is consistent with that reported by Houser *et al*. on diabetes and hypertension in Congolese church staff, which found that religious leaders were more frequently affected by these two chronic diseases [44]. This association could be explained by the generally sedentary nature of these occupations, psychosocial stress related to pedagogical and pastoral responsibilities, and certain eating habits observed during community activities [45].

However, this association observed in the univariate analysis was not confirmed in the multivariate analysis, suggesting that the observed effect was likely influenced by confounding factors, notably age, abdominal obesity, overall physical activity level, and socioeconomic status. Thus, being a teacher or religious leader was not an independent risk factor for diabetes in the studied population. Furthermore, multivariate analysis revealed a significant association between diabetes and the professions of farmer and mason's assistant. At first glance, this result appears paradoxical, as these activities are generally considered protective against diabetes because of their high physical intensity, which improves insulin sensitivity and promotes glycaemic control [46]. However, several plausible epidemiological mechanisms may explain these observations. First, a high level of physical activity is not always sufficient to compensate for the harmful effects of an unbalanced diet on health. Excessive consumption of refined carbohydrates, saturated fats, and low intake of fruits and vegetables remain independent risk factors for diabetes, even in physically active individuals [47]. Second, harmful alcohol consumption and smoking, which are frequently reported in some physically demanding professions, are aggravating factors for metabolic syndrome risk. Alcohol consumption is associated with insulin resistance, abdominal obesity, and glycaemic disorders [48], whereas smoking promotes chronic inflammation and endothelial dysfunction, thereby increasing the risk of type 2 diabetes [49]. Third, the low socioeconomic status of farmers and masons' assistants often find themselves constitutes a major structural determinant of diabetes. Precariousness limits access to healthy food, healthcare services, and early screening, leading to delayed use of healthcare. Numerous studies have highlighted a close association between low socioeconomic status and an increased prevalence of diabetes, particularly in low- and middle-income countries [9].

These results highlight the importance of social determinants of health in the occurrence of diabetes, beyond the level of physical activity. They suggested that in the context of Bunia, simultaneous exposure to unhealthy diets, addictive behaviours, and socioeconomic insecurity could negate the potential protective effects of occupational physical activity. These findings advocate for targeted preventive interventions, not only focused on promoting physical activity but also on improving diet quality, combating alcoholism and smoking, and reducing social health inequalities, particularly among manual workers.

This study had several strengths. The large sample size ensured good precision of the estimates. Geographical representativeness was ensured by including 12 neighbourhoods with urban, semi-urban, and rural characteristics. Data collection by qualified investigators and the use of a random sampling procedure were also major assets. Moreover, this is the first study on the prevalence and associated factors of diabetes in the city of Bunia and Ituri Province.

However, some limitations must be considered. The cross-sectional nature of the study does not allow for the establishment of a causal relationship between the associated factors and the occurrence of diabetes. Furthermore, the diagnosis was based on fasting capillary blood glucose levels without additional investigations such as HbA1c or oral glucose tolerance tests, which could affect the actual estimation of prevalence.

Despite these limitations, this study provides essential epidemiological data to guide public health policy in Bunia. This highlights the need to strengthen prevention strategies, early screening, promote physical activity, combat sedentary lifestyles, and manage individuals with abdominal obesity and a family history of diabetes.

## Conclusion

This study revealed a prevalence of diabetes mellitus of 6.31% among adults in Bunia, with 1.6% of cases newly diagnosed during the survey period. The main factors associated with diabetes were older age, sedentary lifestyle, abdominal obesity, residence in semi-urban areas, family history of diabetes, and low physical activity. Conversely, moderate salt consumption and low-stress levels were associated with a lower prevalence. These results highlight the importance of targeted prevention strategies, including the promotion of physical activity, weight management, and raising awareness of risk factors, to curb the rise of diabetes among adults in Bunia.

### What is known about this topic

- Diabetes is a growing health challenge in sub-Saharan Africa; data in the Democratic Republic of the Congo (DRC) are scarce and mainly originate from hospitals; however, community-level evidence is limited.

### What this study adds

- First community-based estimate of diabetes prevalence in Bunia, Democratic Republic of the Congo;
- Identification of local risk factors: abdominal obesity, sedentary lifestyle, and residence in semi-urban areas;
- The protective factors included moderate salt intake, no family history of diabetes, and low stress levels.
